# Prostaglandin E_2_ Pathway Is Dysregulated in Gastric Adenocarcinoma in a Caucasian Population

**DOI:** 10.3390/ijms21207680

**Published:** 2020-10-16

**Authors:** Catarina Lopes, Carina Pereira, Mónica Farinha, Rui Medeiros, Mário Dinis-Ribeiro

**Affiliations:** 1 Molecular Oncology and Viral Pathology Group, IPO Porto Research Center (CI-IPOP), Portuguese Oncology Institute of Porto (IPO Porto), 4200-072 Porto, Portugal; catarina.p.lopes@ipoporto.min-saude.pt (C.L.); ruimedei@ipoporto.min-saude.pt (R.M.); 2 CINTESIS–Center for Health Technology and Services Research, University of Porto, 4200-450 Porto, Portugal; mdinisribeiro@gmail.com; 3 Pathology Department, Portuguese Institute of Oncology, 4200-072 Porto, Portugal; monicadfarinha@gmail.com; 4 Research Department of the Portuguese League Against Cancer-North (LPCC-NRNorte), Estrada da Circunvalação 6657, 4200-177 Porto, Portugal; 5 Gastroenterology Department, Portuguese Institute of Oncology, 4200-072 Porto, Portugal

**Keywords:** gastric cancer, mRNA expression, prostaglandin E, prostaglandin-endoperoxide synthase 2, hydroxyprostaglandin dehydrogenase 15-(NAD), solute carrier organic anion transporter family member 2A1, ATP-binding cassette subfamily C member 4

## Abstract

Gastric cancer (GC) represents the third leading cause of cancer-related deaths worldwide. The levels of prostaglandin E_2_, a key player in the hallmarks of cancer, are mainly regulated by prostaglandin-endoperoxide synthase 2 (PTGS2) and ATP-binding cassette subfamily C member 4 (ABCC4), involved in its synthesis and exportation, respectively, and 15-hydroxyprostaglandin dehydrogenase (15-PGDH) and solute carrier organic anion transporter family member 2A1 (SLCO2A1), responsible for its inactivation. Even though there are distinct molecular signatures across ethnic populations, most published studies focus on Asian populations. Our main aim was to explore the genetic expression of the aforementioned molecules in a Caucasian population. 94 “Normal” and 89 tumoral formalin-fixed paraffin-embedded (FFPE) samples from GC patients were used to assess the mRNA expression of *PTGS2*, *ABCC4*, hydroxyprostaglandin dehydrogenase 15-(NAD) (*HPGD*), *SLCO2A1* by Real-Time PCR. We found an upregulation for the *PTGS2* gene mean factor of 2.51 and a downregulation for the *HPGD* and *SLCO2A1* genes (mean factor of 0.10 and 0.37, respectively) in tumorous mucosa in a gender-independent manner. In females, we observed an *ABCC4* downregulation and a *PTGS2* mRNA upregulation compared to males in tumoral mucosa (mean factor of 0.61 and 1.64, respectively). We reported dysregulation of the inflammation triggered PGE_2_ pathway in a Caucasian population with an intermediate risk for GC, which might highlight the applicability of aspirin in the treatment of GC patients.

## 1. Introduction

Despite its declining incidence rates, gastric cancer (GC) is still the third most common cause of cancer-related deaths worldwide [1]. Most gastric cancers are adenocarcinomas, which are histologically divided into diffuse and intestinal. The latter is the most common histotype and its geographical distribution overlaps that of *Helicobacter pylori* (*H. pylori*), a causative agent of GC [2]. *H. pylori* is classified as a class I carcinogen by the World Health Organization (WHO) and is involved in the chronic inflammation that likely initiates the multistep progression of intestinal-type GC [3]. This bacterium is also involved in the increase of prostaglandin-endoperoxide synthase 2 (PTGS2, also known as cyclooxygenase-2 (COX-2)) expression within premalignant and malignant gastric lesions [3].

PTGS2 is a key enzyme in prostaglandin (PG) synthesis, namely in PGE_2_ production, and is inhibited by non-steroidal anti-inflammatory drugs (NSAIDs), such as aspirin [4]. PGE_2_ is known to be involved in tumor progression and its effects appear to affect virtually all hallmarks of cancer, namely proliferation, angiogenesis, and migration [4]. PGE_2_ levels depend on synthesis and degradation rates, regulated by PTGS2 and 15-hydroxyprostaglandin dehydrogenase (15-PGDH), respectively [5]. Moreover, the solute carrier organic anion transporter family member 2A1 (SLCO2A1, also known as prostaglandin transporter (PGT)) and ATP-binding cassette subfamily C member 4 (ABCC4, also known as multidrug resistance-associated protein 4 (MRP4)) mediate the PGE_2_ transport in and out of the cell, respectively [5]. 

PTGS2 is the most extensively studied protein of the PGE_2_ pathway, both in GC [6], although most studies focus on Asian populations, and other malignancies, such as liver, esophageal, colorectal [7], and pancreatic cancers, where it is overexpressed compared to normal tissue [4]. 15-PGDH is downregulated in several types of cancer and acts as a tumor suppressor [8,9]. Regarding PGE_2_ transporters, there is a lack of information, but SLCO2A1 downregulation and/or ABCC4 overexpression have been found in some cancers [10]. However, some results are controversial, especially the ones related to SLCO2A1 [11,12].

Even though a study by Kochel et al. [13] reports both PTGS2 and ABCC4 upregulation and 15-PGDH and SLCO2A1 downregulation in breast cancer, no studies address the expression of these four proteins in GC. Since most studies focus on PTGS2 and were reported in Asian populations, the main objective of this study was to explore the genetic expression of *PTGS2*, *ABCC4*, Hydroxyprostaglandin dehydrogenase 15-(NAD) (*HPGD*), and *SLCO2A1*, the genes encoding the proteins mentioned above, in a European country.

## 2. Results

**Figure 1:**
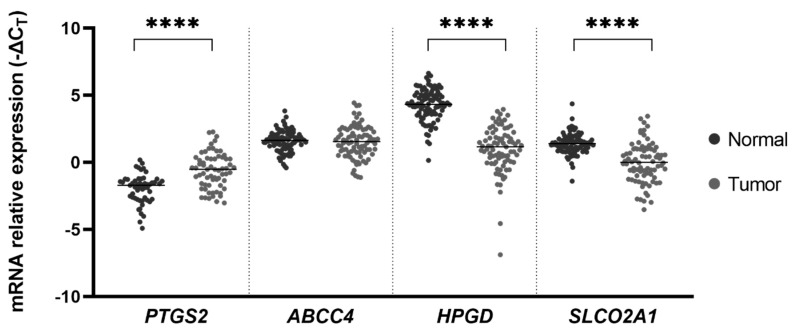
mRNA expression of the prostaglandin E_2_ (PGE_2_) pathway in gastric cancer (GC). prostaglandin-endoperoxide synthase 2 (*PTGS2*) is upregulated in tumor samples compared to normal samples by a mean factor of 2.51, whereas hydroxyprostaglandin dehydrogenase 15-(NAD) (*HPGD*) and solute carrier organic anion transporter family member 2A1 (*SLCO2A1*) are downregulated in tumorous mucosa by a mean factor of 0.10 and 0.37, respectively. Lines represent median values of expression. **** *p* < 0.0001.

RNA was successfully extracted from a total of 94 “normal”-appearing mucosa samples and 89 tumorous mucosa samples. Overall, the *PTGS2* gene was found to be overexpressed in tumor samples compared to normal mucosa (−1.95 ± 0.15 vs. −0.63 ± 0.16; *p* < 0.0001), leading to a 2.51- fold increase in mRNA expression, as can be observed in Figure 1. We found no statistically significant difference in *ABCC4* mRNA expression (1.59 ± 0.79 in normal vs. 1.52 ± 0.12 in tumoral samples; *p* = 0.822). On the other hand, the *HPGD* and *SLCO2A1* genes were found to be downregulated in GC mucosa by a mean factor of 0.10 and 0.37, respectively (4.27 ± 0.12 and 1.40 ± 0.08 in normal samples vs. 0.97 ± 0.19 and −0.02 ± 0.17, respectively; *p* < 0.0001 for both genes). 

Similarly, the expression of these four genes did not differ across early (I and II) and advanced (III and IV) stages of the disease (Figure S1).

**Figure 2:**
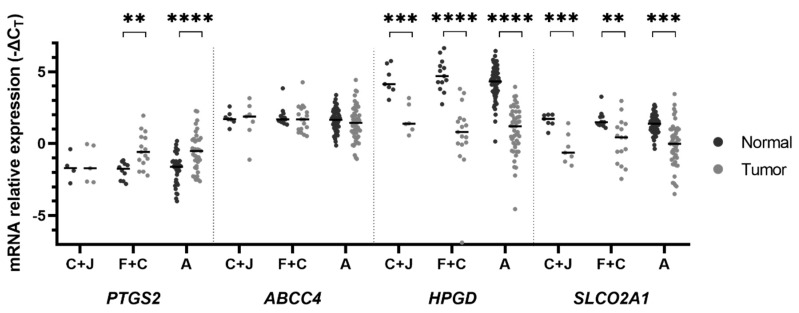
mRNA expression according to localization. *PTGS2* is upregulated in tumor samples by a mean factor of 2.68 and 2.40 in F + C and A, respectively. *HPGD* and *SLCO2A1* genes are downregulated in tumor samples compared to “normal”-appearing mucosa samples by a mean factor of 0.15 and 0.28, 0.14, and 0.36, 0.10, and 0.36 in C + J, F + C, and A, respectively. Lines represent median values of expression. ** *p* < 0.01; *** *p* < 0.001; **** *p* < 0.0001. C + J: Cardia and gastroesophageal junction; F + C: Fundus and corpus; A: Antrum.

We then evaluated the expression profile of the PGE_2_ pathway across different regions of the stomach. In cardia and gastroesophageal junction, we found a downregulation of the *HPGD* and *SLCO2A1* genes in tumorous mucosa (1.70 ± 0.42 vs. 4.42 ± 0.37 in normal mucosa, *p* = 0.0005 for *HPGD* gene; −0.35 ± 0.40 vs. 1.61 ± 0.18 in normal mucosa, *p* = 0.0007 for *SLCO2A1* gene), as displayed in Figure 2. The fundus and corpus and the antrum and transition corpus-antrum demonstrated similar patterns. We found a 2.68-fold increased *PTGS2* mRNA in tumoral mucosa in the fundus and corpus, and a 2.40-fold increase in the antrum and transition (−0.47 ± 0.31 vs. −1.90 ± 0.19 in normal samples of the fundus and corpus, *p* = 0.0024; −0.55 ± 0.21 vs. −1.82 ± 0.18 in normal samples of the antrum and corpus-antrum transition, *p* < 0.0001). Consistent with the overall analysis, a downregulation of *HPGD* and *SLCO2A1* was observed in GC located at the fundus and corpus and antrum and the corpus-antrum transition (0.62 ± 0.58 vs. 4.78 ± 0.31 in normal samples in the fundus and corpus, *p* < 0.0001; 0.93 ± 0.23 vs. 4.25 ± 0.14 in the antrum and transition corpus-antrum, *p* < 0.0001, for the *HPGD* gene and 0.19 ± 0.38 vs. 1.64 ± 0.16 in normal-appearing mucosa of the fundus and corpus, *p* = 0.0021; −0.12 ± 0.22 vs. 1.36 ± 0.08 in the antrum and transition corpus-antrum, *p* < 0.0001 for the *SLCO2A1* gene). Concerning incisura angularis, we only had mRNA expression data from three samples in each histological type and no statistically significant differences were found (data not shown).

**Figure 3:**
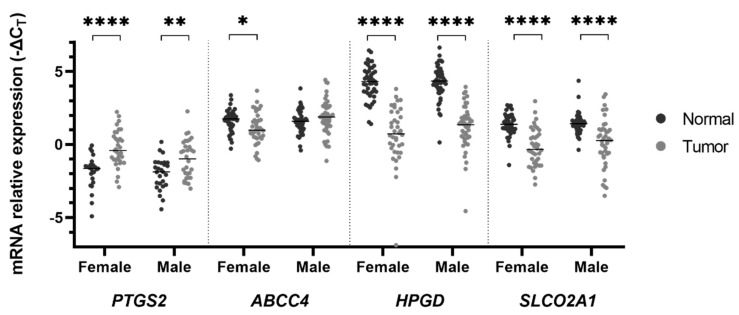
mRNA expression according to gender. *PTGS2* is upregulated in tumor samples by a mean factor of 2.64 in females and 2.05 in males, whereas *HPGD* and *SLCO2A1* genes are downregulated compared to “normal”-appearing mucosa samples by a mean factor of 0.08 and 0.34 (females) and 0.12 and 0.41 (males), respectively. The *ABCC4* gene is also downregulated by a mean factor of 0.71 in tumoral samples in females. Lines represent median values of expression. * *p* < 0.05; ** *p* < 0.01; **** *p* < 0.0001.

A similar expression pattern was observed among males and females (Figure 3), although more noticeably in the latter group. Interestingly, the females presented a statistically significant decrease in *ABCC4* mRNA expression in tumor samples (1.13 ± 0.17 vs. 1.63 ± 0.12 in normal mucosa, *p* = 0.025) by a mean factor of 0.71, which was not observed in males. When we compared the mRNA expression of these genes between genders in tumorous mucosa, we found an *ABCC4* downregulation in females by a mean factor of 0.61 (1.13 ± 0.17 vs. 1.85 ± 1.17 in males, *p* = 0.038) and a *PTGS2* upregulation by a mean factor of 1.64 (−0.28 ± 0.22 vs. −0.99 ± 0.23 in males, *p* = 0.028).

**Figure 4:**
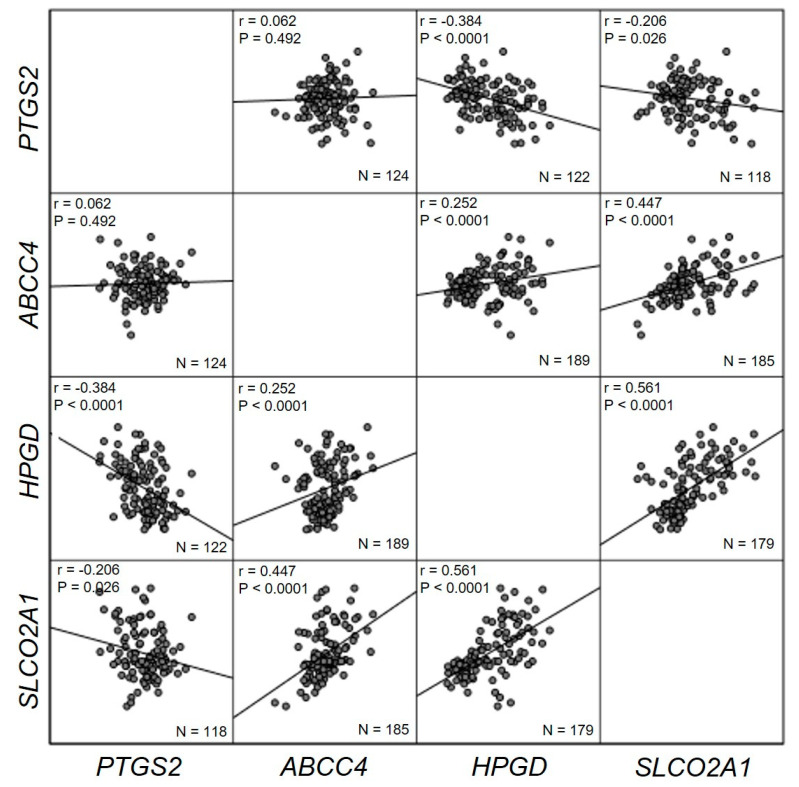
Pearson’s correlation coefficient test. *PTGS2* mRNA expression is negatively correlated with *HPGD* and *SLCO2A1*. On the other hand, there is a positive correlation between the expression of *ABCC4*, *HPGD*, and *SLCO2A1*. No significant correlation was found between *PTGS2* expression and *ABCC4*. Values were considered statistically significant at *p* < 0.05.

Pearson’s correlation coefficient test was computed to assess the relationship between the mRNA expression of the different PGE_2_ pathway genes (Figure 4). Overall, there was a negative correlation between *PTGS2* mRNA expression and the mRNA expression of *HPGD* and *SLCO2A1* (r = −0.384, *n* = 122, *p* ≤ 0.0001; r = −0.206, *n* = 118, *p* = 0.026, respectively). On the other hand, there was a positive correlation between the mRNA expression of the *ABCC4* gene and both the *HPGD* and *SLCO2A1* genes (r = 0.252, *n* = 189, *p* ≤ 0.0001; r = 0.447, *n* = 185, *p* ≤ 0.0001, respectively). The strongest correlation was found between *HPGD* and *SLCO2A1* mRNA expression (r = 0.561, *n* = 179, *p* ≤ 0.0001).

## 3. Discussion

Despite the continuous decrease in incidence worldwide, approximately 75% of GC patients die of the disease, representing the third leading cause of cancer-related deaths [14].

The pleiotropic activities of the PGE_2_/PTGS2 pathway and their effect on cancer progression have been reviewed and explored throughout the years, particularly in colorectal cancer [15,16,17,18]. This pathway involves PGE_2_ synthesis via PTGS2 within the cell and its transport to the extracellular milieu by ABCC4, where it is able to interact with PG receptors (EP1-4) and exert its effects [17,19]. Inversely, SLCO2A1 is responsible for the transport of this PG back into the cell so it can be catabolized and inactivated by 15-PGDH [18]. Dysregulation of this pathway due to PTGS2/ABCC4 overexpression and SLCO2A1/15-PGDH downregulation has been shown to lead to the accumulation of PGE_2_ in the extracellular microenvironment and, therefore, to contribute to its nefarious effects [18,20]. Inhibitors of the COX enzymes, such as aspirin, are potential agents for chemoprevention of GC, but the association of this NSAID with excess bleeding or gastrointestinal damage remains a concern [21].

Considering that the genetic and molecular signatures differ across ethnic populations [22] and the scarcity of published data, here we report, for the first time and to the best of our knowledge, the dysregulation of several PGE_2_ pathway-related genes in Caucasian patients diagnosed with GC.

Our results show an increase in *PTGS2* mRNA expression in tumoral samples compared to the normal mucosa and, on the other hand, a downregulation of the *HPGD* and *SLCO2A1* genes, in a gender-independent manner. The same pattern was observed across the different regions of the stomach under study, with the exception of the cardia and gastroesophageal junction, where we did not find a statistically significant difference in the *PTGS2* mRNA expression between cancerous and normal mucosa. ABCC4’s role as a drug transporter, which may also contribute to cancer progression, has been explored in a variety of diseases. In opposition to what was previously reported in other cancer models [10,23,24], no differences were found in the mRNA levels of the *ABCC4* gene. Interestingly, when we compared the mRNA values in the tumoral mucosa between males and females, we found a decrease in *ABCC4* mRNA expression in females in our population. Previous findings revealed an overexpression of *ABCC4* mRNA levels in female mice kidneys and liver [25]. In the former organ of those rodents, ABCC4 appeared to be repressed by the male hormone 5α-dihydroxytestosterone and by male-pattern growth-hormone secretion [26]. Further studies are necessary to understand if there are other factors contributing to the difference in *ABCC4* mRNA expression we observed between males and females.

PTGS2 is undoubtedly the most studied protein of this pathway, explored in a variety of cancers and found upregulated through a variety of methods (northern blot, immunoblotting, and RT-PCR) [27]. In GC, the majority of studies characterizing this protein expression are reported in Asian populations [28,29,30,31,32,33,34,35,36], with few studies involving North American [37], African [38], and European patients [39,40,41,42]. Overall, in those studies, a similar expression pattern is found between distinct ethnic populations, with a higher PTGS2 expression in tumoral samples compared to normal tissue and an increased expression with the progression of mucosal damage.

The role of 15-PGDH in GC is still somewhat controversial. While some studies report a decreased expression of this protein in gastric malignancies compared to normal gastric tissues, others show no such difference [8,43,44,45,46,47]. It is noteworthy that all these studies were performed in either Chinese or Korean populations. Moreover, 15-PGDH has been regarded as a tumor suppressor by some reports and has been associated with the development of gastric carcinoma by inducing apoptosis and cell cycle arrest [8,44,45,46]. The correlation between 15-PGDH expression and some pathological findings has also been controversial. Some authors defend no correlation between the expression of this enzyme and the tumor-node-metastasis stage, vascular invasion, and tumor histologic type [45,48], whereas a study by Seo et al. [47] reports a significant correlation between 15-PGDH expression and the T and N stage, pathologic type, metastasis, vascular, lymphatic, and perineural invasion, and palliative gastrectomy. In addition, that study associates the expression of 15-PGDH with five-year gastric-cancer-specific survival, but it does not classify it as an independent prognostic factor [47]. On the other hand, Tatsuwaki et al. [49] performed a multivariate analysis and concluded that reduction of 15-PGDH expression could, in fact, be an independent predictor of poor survival and it was correlated with differentiation, disease stage, which was not observed in our study, and prognosis. Furthermore, *H. pylori* infection appears to promote gastric carcinogenesis by modulating both *HPGD* and *PTGS2* mRNA expression and protein synthesis [31,50]. 

As mentioned previously, not much is known about the PGE_2_ transporters in GC. Reduced SLCO2A1 expression has been associated with increasing PGE_2_ levels in the tumor microenvironment and, consequently, with tumor angiogenesis in GC [51]. Moreover, colocalization of this transporter and the PGE_2_ receptor EP4 has been detected in the mucosa of both normal stomach and gastric carcinoma, suggesting a role of SLCO2A1 in the PGE_2_-mediated cellular effects [52]. In the same study, by Bujok et al. [52], a higher expression of the protein was found in GC tissue compared to normal tissue, but with no statistical significance. Takeda et al. [53] were the first to identify SLCO2A1 expression as an independent predictor of poor prognosis in patients with GC. In this study, reduction of the transporter expression correlated with increased tumor angiogenesis and its suppression by specific small interfering RNA (siRNA) promoted the production of vascular endothelial growth factor (VEGF), a mediator of angiogenesis, induced by PGE_2_ [53]. Furthermore, the immunohistochemical staining showed a diffuse SLCO2A1 expression in normal gland epithelial cells of the stomach, similar to other expression patterns reported previously in normal intestinal cells and suggesting a strict regulation of PGE_2_ concentration to maintain cellular homeostasis [53]. The authors presume that this homeostasis is impaired in gastric tumors due to the negative regulation of SLCO2A1 and, consequently, the negative regulation of PGE_2_ degradation, resulting in the enhancement of PGE_2_ signaling and gastric tumorigenesis [53]. Contrary to those reports, a study by Nakanishi et al. [54] suggests an association between higher SLCO2A1 expression in colorectal cancer and poor prognosis. The authors indicate a likely promotion of tumorigenesis by PGE_2_ uptake into the endothelial cells via this transporter [54]. In ovarian cancer, both increased and reduced levels of SLCO2A1 have been reported [55,56]. In sum, in this study, we found a dysregulation in the PGE_2_ pathway in Caucasian GC patients. *PTGS2* mRNA expression was found to be negatively correlated with *HPGD* and *SLCO2A1* expression, whereas *ABCC4*, *HPGD*, and *SLCO2A1* expressions were positively correlated. Despite a similar pattern reported for *PTGS2*, *HPGD*, and *SLCO2A1* genes, the heterogeneous expression of *ABCC4* in GC across ethnicities might contribute to explain the differences observed in GC incidence worldwide.

There have been reports on distinct biological and transcriptomic signatures between cancer, normal adjacent mucosa, and healthy tissues [57,58]. Even though we performed macrodissection of normal tissue distant from the tumoral cells whenever feasible, it is possible that the use of these samples as controls could lead to suboptimal results.

Currently, in countries with moderate to low incidences, such as Portugal, mass screening such as the one observed in Japan and Korea is unwarranted [59]. Nevertheless, the stratification of the population by GC risk might allow a personalized screening/surveillance, namely by targeted screening, optimization of surveillance intervals, and even selection for chemoprevention. The identification of mRNA transcripts associated with cancer development is a step towards personalized medicine. Furthermore, we reported an increased *PTGS2* mRNA expression in tumoral samples, hence, it is plausible that if one could identify individuals with a higher probability of overexpressing PTGS2, these might be targeted towards NSAIDs-base GC chemopreventive strategies.

## 4. Materials and Methods 

### 4.1. Patient Samples

This study was approved by the Ethics Committee at the Instituto Português de Oncologia do Porto (IPO-Porto) on 15 December 2016 (CES.314/016). Furthermore, the study protocol conforms to the Declaration of Helsinki ethical guidelines as reflected in a priori approval by the Research Centre at IPO-Porto (CI-IPOP 48-2016). After revision of the histopathological database from the Pathology department at IPO-Porto, 121 patients with histological confirmation of intestinal-type GC, diagnosed between May 2012 and December 2015, were randomly selected from a consecutive series of cases based on the availability of formalin-fixed paraffin-embedded (FFPE) samples. Written informed consent was previously given by each patient authorizing the inclusion of surplus tissues in the Institute’s tumors’ biobank for future use in biomedical research. The description of the population is summarized in Table 1. Overall, the median age of the participants was 70 years with males representing 50% of cases. Most tumors were located in the antrum and corpus-antrum transition (60%) and presented moderately differentiated cells (56%). Concerning tumor staging, nearly half the GC patients were diagnosed in early stages of the disease (49% for stages I and II vs. 51% for stages III and IV). No differences in the demographic and tumor characteristics variables were observed between the participants in this study and the overall population of GC patients.

Medical records were reviewed to extract the clinicopathological variables, such as localization, stage, and tumor grade. All tumors were restaged according to the eighth edition of the AJCC (American Joint Committee on Cancer) Cancer Staging Manual [60].

### 4.2. Nucleic Acid Isolation and Quantification

RNA was extracted from FFPE samples using the AllPrep DNA/RNA FFPE Kit (Qiagen, Hilden, Germany) according to the manufacturer’s instructions. A 3 µm section stained with hematoxylin and eosin (H&E) was histopathologically characterized at the Pathology Department at IPO-Porto and the number of 10 µm sections used for nucleic acid extraction varied from two to six, depending on the size of the limited area (up to 6 cm^2^) enriched in “normal”, distant from the tumor whenever possible, or tumoral cells. Using a sterile single-use scalpel, the area was macrodissected into a 1.5 mL microcentrifuge tube containing 1 mL of deparaffination reagent D-limonene (Santa Cruz, Dallas, TX, USA) by scratching. The kit instructions were followed throughout the remaining procedure.

The resulting RNA was quantified using the NanoDrop^TM^ Lite Spectrophotometer (Thermo Fisher Scientific, Waltham, MA, USA), its quality was assessed by measuring the optical density (OD) 260/280 ratio, and it was kept at −20 °C until further processing.

### 4.3. Reverse Transcription Reaction

Approximately one hundred samples of “normal”-appearing mucosa and one hundred samples of tumoral mucosa were included in the study. Complementary DNA (cDNA) was synthesized from up to 2 µg of RNA using the High Capacity cDNA Reverse Transcription (RT) kit (Thermo Fisher Scientific, Waltham, MA, USA) following the manufacturer’s instructions. Briefly, in a 20 µL reaction mix, 2.0 µL of 10X RT Buffer, 0.8 µL of 25X dNTP Mix (100 mM), 2.0 µL of 10X RT Random Primers, 1.0 µL of MultiScribe Reverse Transcriptase, 4.2 µL of nuclease-free water, and 10.0 µL of RNA were used. The RT conditions were as follows: Annealing at 25 °C for 10 min, DNA polymerization at 37 °C for 120 min, and enzyme deactivation at 85 °C for 5 min. 

All RT reactions included one no-template negative control. Moreover, 1 µL containing 1 µg of the QPCR Human Reference Total RNA, part of the Absolutely RNA FFPE Kit (Agilent, Santa Clara, CA, USA), was used as a positive control to monitor the quality of the RT.

### 4.4. Real-Time PCR

cDNA amplification by Real-Time PCR was performed using a StepOne Plus Real-Time PCR system (Applied Biosystems, Foster City, CA, USA). In a 10 µL reaction mix, 5.0 µL of TaqMan^®^ Gene Expression Master Mix (Applied Biosystems, Foster City, CA, USA), 0.5 µL of TaqMan^®^ Gene Expression Assay (Applied Biosystems, Foster City, CA, USA), and 20 ng of cDNA template were used. 

The gene expression assays used to measure the mRNA expression of the *PTGS2*, *HPGD*, *ABCC4*, and *SLCO2A1* genes were Hs00153133_m1, Hs00168359_m1, Hs00988717_m1, and Hs01114926_m1 (Applied Biosystems, Foster City, CA, USA), respectively. All assays underwent the following thermal cycling conditions: 50 °C for 2 min, 95 °C for 10 min, and 40 cycles of 95 °C for 15 s and 60 °C for 1 min. They were validated to determine the efficiency of the amplification reaction and their limit of detection using a 1:2 dilution series with 7 dilution steps. As a result, efficiencies between 90–105% and sensitivity above 6.25 ng were reported for all gene expression assays used.

A panel of six reference genes (*B2M*, *HPRT1*, *RPL29*, *PPIA*, *IPO8*, and *GUSB*) was selected, after reviewing the literature on gastrointestinal cancers, to determine the most adequate for this experiment using the NormFinder and geNorm softwares (25, 26). The gene expression was assessed using the Hs00187842_m1, Hs02800695_m1, Hs00988959_gH, Hs99999904_m1, Hs00914057_m1, and Hs99999908_m1 TaqMan^®^ gene expression assays (Applied Biosystems, Foster City, CA, USA), respectively. *HPRT1* and *IPO8* were selected as the most stable combination of genes to normalize our results, with a stability value of 0.094 (determined by NormFinder). The stability values of all the genes are displayed in Table S1.

Triplicates were used for mRNA quantification using real-time PCR and replicates with a standard deviation (SD) superior to 30% of 1 cycle threshold (C_T_) were excluded. For each sample, all target and reference genes were amplified in the same plate. Additionally, one positive control from the RT reaction and three no template negative controls were included. The endpoint of the real-time PCR was the C_T_ determined as the average value from three independent reactions.

### 4.5. Statistical Analysis

The relative mRNA expression was expressed as the difference between C_TS_ of the amplification curves of the target genes (*PTGS2*, *ABCC4*, *HPGD*, and *SLCO2A1*) and the reference genes (−∆C_T_). The expression fold-change was calculated following the Livak method (2^−∆∆Ct^) [61].

Statistical analysis was performed using the computer software *IBM^®^ SPSS^®^ Statistics* (IBM Corp., Armonk, NY, USA) version 26.0 for Windows. Additionally, GraphPad Prism version 8.00 for Windows was used to obtain graphical designs. Student’s t-test was performed to compare mean values between values (normal vs. tumor, female vs. male) and the correspondent nonparametric tests were applied when appropriate. Values were considered statistically significant at *p* < 0.05.

All authors had access to the study data and have reviewed and approved the final manuscript.

## Figures and Tables

**Table 1:** Description of the participants.

	Cases
**Demographics (*n* = 121)**	
Age (years)	
Mean ± sd	69.49 ± 0.91
Median (min-max)	70 (50–89)
Sex, *n* (%)	
Male	60 (50)
Female	61 (50)
**Tumor characteristics (*n* = 89)**	
Tumor location, *n* (%)	
Cardia and GEJ	7 (8)
Fundus and corpus	18 (20)
Antrum and corpus-antrum transition	53 (60)
Angularis incisura	3 (3)
Others *	8 (9)
Grade, *n* (%)	
Well-differentiated	11 (13)
Moderately differentiated	56 (63)
Poorly differentiated	19 (21)
Cannot be assessed	3 (3)
Stage, *n* (%)	
I-II	44 (49)
III-IV	45 (51)
Synchronous tumors, *n* (%)	
Yes	7 (8)
No	82 (92)

* Including tumors that occupy more than one location and tumors of the gastric stump. For synchronous tumors, the most advanced lesion was considered in the characterization.

## Supplementary Materials

The following are available online at https://www.mdpi.com/1422-0067/21/20/7680/s1, Figure S1. mRNA expression across GC stages. Table S1. Stability expression values for normalization of GC and GN samples from NormFinder and GeNorm softwares.

## Abbreviations

15-PGDH	15-hydroxyprostaglandin dehydrogenase
*ABCC4*	ATP-binding cassette subfamily C member 4
AJCC	American Joint Committee on Cancer
*B2M*	Beta-2-microglobulin
cDNA	Complementary deoxyribonucleic acid
COX-2	Cyclooxygenase-2
C_T_	Cycle threshold
EP	Prostaglandin E_2_ receptor
FFPE	Formalin-fixed paraffin-embedded
GC	Gastric cancer
*GUSB*	Glucuronidase beta
H&E	Hematoxylin & eosin
*H. pylori*	*Helicobacter pylori*
*HPGD*	Hydroxyprostaglandin dehydrogenase 15-(NAD)
*HPRT1*	Hypoxanthine phosphoribosyltransferase 1
IPO8	Importin 8
mRNA	Messenger ribonucleic acid
MRP4	Multidrug resistance protein 4
NSAIDs	Non-steroidal anti-inflammatory drugs
OD	Optical density
PCR	Polymerase chain reaction
PGE_2_	Prostaglandin E_2_
PGT	Prostaglandin transporter
*PPIA*	Peptidylprolyl isomerase A
*PTGS2*	Prostaglandin-endoperoxide synthase 2
*RPL29*	Ribosomal protein L29
RT	Reverse transcription
SD	Standard deviation
siRNA	Small interfering ribonucleic acid
*SLCO2A1*	Solute carrier organic anion transporter family member 2A1
SPSS	Statistical package for the social sciences
VEGF	Vascular endothelial growth factor
WHO	World Health Organization
