# Supplementary material for: Prostaglandin E2 Pathway Is Dysregulated in Gastric Adenocarcinoma in a Caucasian Population
Source: Int J Mol Sci. 2020 Oct 16;21(20):7680. doi: 10.3390/ijms21207680 (PMC7589882; doi:10.3390/ijms21207680)
Supplement: Supplementary file 1 [file ijms-21-07680-s001.pdf]

SUPPLEMENTAL DATA

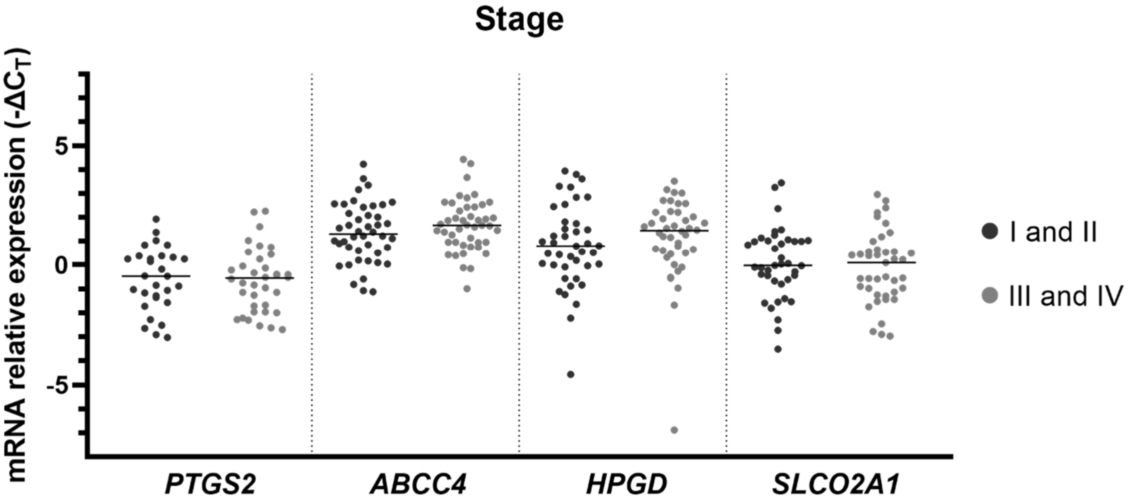

Figure S1. mRNA expression across GC stages. No statistically significant differences are found in the mRNA expression of the *PTGS2*, *ABCC4*, *HPGD*, and *SLCO2A1* genes across GC stages. Lines represent median values of expression.

Table S1. Stability expression values for normalization of GC and GN samples from NormFinder and GeNorm softwares.

**Gene name**	**Algorithm**	**Intragroup variation (*M*)**		**Intergroup variation (*M*)**
		GC	GN	
B2M	NormFinder	0.140	0.234	0.267
	GeNorm	0.648	0.799	0.761
HPRT1	NormFinder	0.027	0.060	0.125
	GeNorm	0.492	0.601	**0.541**
RPL29	NormFinder	0.098	0.045	0.244
	GeNorm	0.595	0.584	0.647
PPIA	NormFinder	0.044	0.099	0.451
	GeNorm	0.522	0.627	0.837
IPO8	NormFinder	0.032	0.101	**0.106**
	GeNorm	0.496	0.634	0.560
GUSB	NormFinder	0.146	0.118	0.185
	GeNorm	0.661	0.678	0.687

GC: tumorous mucosa samples; GN: normal mucosa samples.

Values in **bold** correspond to the lowest *M* values for each algorithm.
